# *Lactobacillus reuteri* and *Enterococcus faecium* from Poultry Gut Reduce Mucin Adhesion and Biofilm Formation of Cephalosporin and Fluoroquinolone-Resistant *Salmonella enterica*

**DOI:** 10.3390/ani11123435

**Published:** 2021-12-01

**Authors:** Abubakar Siddique, Sara Azim, Amjad Ali, Fazal Adnan, Maryum Arif, Muhammad Imran, Erika Ganda, Abdur Rahman

**Affiliations:** 1 Atta Ur Rahman School of Applied Biosciences (ASAB), National University of Sciences and Technology (NUST), Islamabad 44000, Pakistan; mabubakar.asab@asab.nust.edu.pk (A.S.); saraazim94@gmail.com (S.A.); amjad.ali@asab.nust.edu.pk (A.A.); adnanfazal@asab.nust.edu.pk (F.A.); 2 Institute of Food and Nutritional Sciences, PMAS-Arid Agriculture University, Rawalpindi 46000, Pakistan; mariumrajaz@gmail.com; 3 Department of Biosciences, Faculty of Sciences, COMSATS University Islamabad (CUI), Park Road, Islamabad 45550, Pakistan; m.imran@comsats.edu.pk; 4 Department of Animal Sciences, The Huck Institute of Life Sciences, Penn State University, University Park, PA 16802, USA; ganda@psu.edu

**Keywords:** poultry, *Lactobacillus reuteri*, *Enterococcus faecium*, non-typhoidal *Salmonella*, multidrug resistance, probiotics

## Abstract

**Simple Summary:**

Enteric infections, such as *Salmonella* spp., are common in the poultry sector. Even though the European Union has banned the use of growth-promoting antibiotics; many countries continue to use these synthetic medications, leading to the emergence of antibiotic resistance (especially cephalosporin and fluoroquinolones) in non-typhoidal *Salmonella*, limiting treatment options. Probiotics are beneficial bacteria that reside in the intestine and help improve the host’s health; they are also one of the most popular antibiotic alternatives. As a result, we set out to collect lactic acid bacteria from the poultry gut that had never been fed a medicated diet and conduct in vitro probiotic studies. *L. reuteri* PFS4, *E. faecium* PFS13, and *E. faecium* PFS14 were screened as potential probiotic candidates. The obtained strains show good aggregation, mucin adherence, antibiofilm, and anti-salmonella activities. More research is now being conducted to determine the strain’s efficacy in commercial poultry.

**Abstract:**

Non-typhoidal *Salmonella* (NTS) can cause infection in poultry, livestock, and humans. Although the use of antimicrobials as feed additives is prohibited, the previous indiscriminate use and poor regulatory oversight in some parts of the world have resulted in increased bacterial resistance to antimicrobials, including cephalosporins and fluoroquinolones, which are among the limited treatment options available against NTS. This study aimed to isolate potential probiotic lactic acid bacteria (LAB) strains from the poultry gut to inhibit fluoroquinolone and cephalosporin resistant MDR *Salmonella* Typhimurium and *S*. Enteritidis. The safety profile of the LAB isolates was evaluated for the hemolytic activity, DNase activity, and antibiotic resistance. Based on the safety results, three possible probiotic LAB candidates for in vitro *Salmonella* control were chosen. Candidate LAB isolates were identified by 16S rDNA sequencing as *Lactobacillus reuteri* PFS4, *Enterococcus faecium* PFS13, and *Enterococcus faecium* PFS14. These strains demonstrated a good tolerance to gastrointestinal-related stresses, including gastric acid, bile, lysozyme, and phenol. In addition, the isolates that were able to auto aggregate had the ability to co-aggregate with MDR *S.* Typhimurium and *S.* Enteritidis. Furthermore, LAB strains competitively reduced the adhesion of pathogens to porcine mucin Type III in co-culture studies. The probiotic combination of the selected LAB isolates inhibited the biofilm formation of *S.* Typhimurium FML15 and *S.* Enteritidis FML18 by 90% and 92%, respectively. In addition, the cell-free supernatant (CFS) of the LAB culture significantly reduced the growth of *Salmonella* in vitro. Thus, *L. reuteri* PFS4, *E. faecium* PFS13, and *E. faecium* PFS 14 are potential probiotics that could be used to control MDR *S*. Typhimurium and *S.* Enteritidis in poultry. Future investigations are required to elucidate the in vivo potential of these probiotic candidates as *Salmonella* control agents in poultry and animal feed.

## 1. Introduction

Non-typhoidal *Salmonella* serovars *S.* Typhimurium and *S.* Enteritidis cause economic losses in poultry by reducing growth and egg production and pose a food safety risk to humans due to potential carcass contamination in slaughterhouses [1]. In fact, poultry products such as meat and eggs have been implicated as a major cause of non-typhoidal *Salmonella* (NTS) in humans [2]. The poultry industry is Pakistan’s most important agricultural activity, contributing 1.3% to the country’s gross domestic product [3]. In addition to its economic importance, the Pakistani poultry sector contributes significantly to efforts to close the gap between the demand and supply of animal protein. However, the poultry industry faces several challenges, including the emergence of multidrug-resistant foodborne pathogens, which can significantly endanger animal and human health and negatively impact economic output [4]. Antibiotics and vaccines are the primary control strategies to combat salmonellosis in poultry farms [5], but ever-increasing antimicrobial resistance results in antibiotics becoming less effective, while vaccine efficacy remains suboptimal [6].

Antibiotics have been widely used as a prophylactic measure and as growth promoters in poultry production [7]. The use of clinically significant antibiotics as feed additives in poultry led to the emergence and spread multi-drug-resistant enteropathogenic bacteria, including *Salmonella* serovars [8,9]. Many studies have reported that multidrug-resistant *Salmonella* serovars *S.* Typhimurium and *S.* Enteritidis had been isolated from poultry and clinical samples [10,11]. Routine monitoring in 2018–2019 revealed that *Salmonella* spp. isolated from animals and food was resistant to ampicillin, tetracyclines, and sulfonamides, similar to what was observed in *Salmonella* isolates reported from human cases during the same time period [12]. Resistance to quinolones was also very high among *Salmonella* spp. recovered from broilers, finishing turkeys, and poultry carcasses/meat during 2018, substantiating the threat these pathogens pose to the poultry industry [13].

Cephalosporins and fluoroquinolones (enrofloxacin and ciprofloxacin) are the drugs of choice for the treatment of *S.* Typhimurium and *S.* Enteritidis caused infections in poultry and humans [14,15]. *S.* Enteritidis, a serovar predominantly associated with poultry, demonstrated increasing trends in resistance in eight countries between 2015 and 2019 [16]. Ciprofloxacin-resistant *Salmonella* spp. was also isolated from human samples in 2019 [17]. Of relevance to Pakistan, rising cephalosporin resistance in *S*. Typhimurium and other *S. enterica* serovars from food samples have also been reported [18]. Due to the widespread problem of antimicrobial resistance to key antibiotics, there is a substantial need for antibiotic alternatives in the control and prevention of *Salmonella* [19]. According to the Organization for Economic Cooperation and Development (OECD), probiotics are a promising alternative therapy to topical antibiotics [20].

Probiotics have been shown to improve host health and to protect the host from enteric bacteria that can cause GIT infections. [21,22]. Probiotics control pathogens through various mechanisms, including producing antimicrobial substances, enhancing mucosal barrier function, competing with pathogens for adhesion sites, and interacting with the host’s immune system [23,24,25]. Probiotics also have the potential to be used in place of antibiotics as growth promoters, further reducing the selective pressure and spread of antimicrobial resistance. To effectively control pathogens and adapt to the host GIT, the source of probiotic bacteria is an essential feature. Thus, the selection of host-specific probiotic strains is critical for optimal probiotic production.

The in vitro characterization of potential probiotics is considered as a practical approach to evaluate isolates against multidrug-resistant (MDR) pathogens [26,27,28]. Competitive inhibition, growth kinetics, and co-aggregation are some of the most used methods to investigate the potential of probiotics for pathogen control. Several in vitro and in vivo studies have provided evidence that probiotic bacteria can help control *Salmonella* in food animals [29,30]. As part of the one health concept, controlling *Salmonella* in food producing animals and food production systems using probiotics could ultimately reduce pathogen spread to humans. Probiotic-mediated control of cephalosporin- and quinolone-resistant NTS could help contain the spread of antimicrobial resistance to other pathogens, further benefiting animals and humans.

Although routinely used as human probiotics, lactic acid bacteria (LAB) isolated from the poultry gut have not been extensively studied as potential probiotics for use in the poultry industry. To the best of our knowledge, very little information on the potential antagonistic activity of LAB against third generation cephalosporin and quinolone resistant NTS is available. The current study aims to screen probiotic LAB isolated from poultry gut for their potential inhibitory effect against MDR *S*. Typhimurium and *S*. Enteritidis of a poultry origin.

## 2. Methodology

### 2.1. Isolation of Lactic Acid Bacteria

Cloacal swab samples were collected from broilers (*n* = 10) in three local poultry farms located in the Islamabad capital territory. The birds’ health was certified by the resident veterinarian before sampling. The birds were reared on an antibiotic-free diet. Samples were immediately stored at 4 °C and were processed within 6 h. The fecal samples were processed as previously described [31]. Three to five suspected lactic acid bacteria colonies were characterized by Gram-staining and catalase testing, followed by microscopic examination. Only Gram-positive, catalase-negative isolates with a rod or coccus shape were selected for further studies.

### 2.2. Test Pathogens

*S*. Typhimurium FML15 and *S*. Enteritidis FML18, previously isolated from poultry feces, were selected as the test pathogens. Both serovars were multi-drug resistant (MDR), and resistance was observed against third generation cephalosporin and quinolone, as characterized in our previous study [32].

### 2.3. Safety Assessments

#### 2.3.1. Hemolytic Activity

The hemolytic activity was determined as described previously [25]. Overnight-grown bacterial cultures were streaked on Blood agar (Oxoid, Hampshire, UK). Agar plates were incubated at 37 °C for 48–72 h and were observed for the formation of any clean (α-hemolysis), greenish (β-hemolysis) hemolytic zones or no zone (γ-hemolysis) around the LAB colonies [33]. *Staphylococcus aureus* was used as a positive control.

#### 2.3.2. DNase Activity

For the DNA degradation activity, bacterial colonies were streaked on DNase agar (Oxoid, Hampshire, UK). The plates were incubated for 48–72 h at 37 °C and were observed for the clear zone of the DNase activity [34]. DNase activity was considered positive in the clear or pinkish zones surrounding the colonies [35]. *S. aureus* was used as a positive control.

#### 2.3.3. Antibiotic Resistance Profiling

Antibiotic resistance profiling of LAB isolates was determined using the disk diffusion method as per CLSI guidelines [36]. The pattern of antibiotic resistance was tested against widely used antibiotics in poultry production and clinical practices. The following 15 antibiotics were tested: Tetracycline 30 µg, Kanamycin 30 µg, Ciprofloxacin 5 µg, Chloramphenicol 30 µg, Gentamicin 10 µg, Amoxicillin/clavulanic acid 30 µg, Vancomycin 30 µg, Rifampicin 10 µg, Meropenem 5 µg, Imipenem 5 µg, Cefepime 10 µg, Cefixime 10 µg, Streptomycin 30 µg, Sulfamethoxazole-Trimethoprim 25 µg, Linezolid 10 µg, Amikacin 10 µg, and Nalidixic acid 10 µg. An overnight-grown 100 µL bacterial suspension was inoculated on MRS and M17 agar plates. Antibiotic discs were then placed on the agar plates and incubated at 37 °C for 24 h. After incubation, the zone of inhibition was measured. The resistance pattern of the LAB isolates was interpreted as follows: a diameter of ≥21 mm indicating a susceptible (S) strain, a diameter of 16–20 mm revealed an intermediate (I) strain, and a diameter ≤15 mm indicating a resistant (R) strain [37].

### 2.4. Identification of the Lactic Acid Bacterial Isolates

The selected lactic acid bacteria were identified by partial sequencing of the 16S rDNA genes. The genomic DNA was extracted using the phenol–chloroform extraction method, as described previously [38]. The DNA was quantified using a nanodrop spectrophotometer (Thermo Scientific) as per the user manual. The extracted DNA was used as a template in PCR targeted at the 16S rRNA gene using primers 9F (GAGTTTGATCCTGGCTCAG) and 1510R (GGCTACCTTGTTACGA), with the following PCR conditions: 1 cycle of 94 °C for 5 min followed by 35 cycles of 94 °C for 30 s, 54 °C for 45 s, and 72 °C for 30 s, and finally 1 cycle of 7 min at 72 °C [39]. The purified PCR products of the selected LAB isolates were sequenced [40]. The strains were identified by comparing the sequences with the GenBank database using the Basic Local Alignment Search Tool [41].

### 2.5. Screening of Probiotic Properties of LAB Isolates

#### 2.5.1. Acid Tolerance

The resistance to a low pH was assessed in vitro to determine the isolates’ ability to withstand passage through the gastric cavity, as described previously [39]. LAB isolates were grown for 24 h at 37 °C in MRS and M17 broth. In addition, 30 µL overnight-grown LAB isolates were suspended in 150 μL of MRS broth and M17 broth adjusted to pH 2.0 by 1M HCl. The cell suspensions were incubated anaerobically at 37 °C for 4 h. After incubation, bacterial growth was monitored by measuring the absorbance at 600 nm. All experiments were performed in triplicate.

#### 2.5.2. Bile Tolerance

Bile tolerance tests for LAB isolates were performed as described [39]. First, 30 μL of overnight grown LAB isolates were suspended in 150 μL of MRS, and M17 broth with 0.3% bile salt (Oxoid, Hampshire, UK) was added to each well, while mixtures without bile salt were used as a control. The cell suspensions were incubated anaerobically at 37 °C for 4 h. Optical density (OD) at 600 nm was measured for monitoring the growth kinetics on a microtiter plate reader (Bio-Rad Laboratories, CA, USA). All of the experiments were performed in triplicate.

#### 2.5.3. Lysozyme Tolerance

The lysozyme tolerance test was performed as described previously [39]. The overnight-grown cultures were harvested by centrifugation at 3500× *g* for 5 min. The bacterial cells were washed with phosphate buffer saline (PBS) and were re-suspended in a sterile PBS solution. The cell suspension (10 µL) was then inoculated into a PBS solution containing lysozyme (100 mg/L). Isolates inoculated in PBS without lysozyme were used as a control. LAB isolates were incubated at 37 °C. OD was measured at 600 nm after 30 and 90 min.

#### 2.5.4. Phenol Tolerance

Gut microbes can break down aromatic amino acids, which result in the formation of phenol, which can inhibit the growth of LAB. Therefore, resistance to phenol tolerance by LAB is vital for their survival in GIT. Overnight-grown bacterial cultures were inoculated in respective broths supplemented with 0.4% phenol. OD was measured at 600 nm [39].

### 2.6. Cell Surface Properties

#### 2.6.1. Auto Aggregation and Co-Aggregation Assay

The aggregation ability of LAB strains was studied as described previously [42]. The overnight grown culture was harvested by centrifugation at 5000× *g* for 20 min at 4 °C, and was washed and then suspended in PBS. Optical densities were adjusted at 0.25 ± 0.05 OD to maintain the number of bacterial cells (10^7^–10^8^ CFU/mL). For auto-aggregation, the OD of the LAB strains was recorded at 0 h and 24 h at 37 °C. The aggregation percentage was expressed as [1 − (A_Time_/A_0_) × 100], where A_Time_ represents the absorbance of the mixture at 24 h and A_0_ represents the absorbance at 0 h (21). The criteria for the auto-aggregation ability determination were (low, 16–35%; medium, 35–50%; and high, 50% and above) [43].

For the co-aggregation assay, bacterial suspensions of LAB strains, as described above, were mixed with an equal volume (4 mL) of the overnight grown cultures of *Salmonella* in a sterile falcon tube; the mixtures were incubated at 37 °C. After 24 h, the OD_620 nm_ was measured, and the percent co-aggregation was calculated as follows: [(*A*_pathog_ + *A*_LAB_)/2 − (*A*_mix_)/(*A*_pathog_ C *A*_LAB_)/2] × 100, where *A*_pathog_ and *A*_LAB_ represent the absorbance in the tubes containing only the pathogen and LAB strain, respectively, and *A*_mix_ represents the absorbance of the mixture at 24 h [42].

#### 2.6.2. Microbial Adhesion to Hydrocarbon Test (MATH)

A bacterial cell surface hydrophobicity assay was performed with some modifications [44]. LAB isolates cultivated in MRS and M17 broth at 37 °C for 24 h were harvested by centrifugation at 8000× *g* for 10 min, and the cell pellet was washed thrice with sterile PBS and was re-suspended in the same media. The optical densities were maintained 1 ± 0.05 at 600 nm. Initially, 3 mL of the bacterial cell suspension was transferred to a sterile falcon tube, followed by the addition of 1 mL of hydrocarbons. Three hydrocarbons were tested in this study, namely: toluene (apolar, aromatic), xylene (apolar, aromatic), and chloroform (polar, aliphatic). The tubes were incubated at 37 °C for 10 min for temperature equilibrium, followed by 15 s vortexing, and were then set at 37 °C for 20 min for the phase separation. The lower aqueous phase was carefully collected in a separate glass tube, and OD_600 nm_ was recorded.

% affinity = (OD_i_ − OD_t_/OD_i_) × 100 (1)

OD_i_ is the initial OD of the cell suspension, and OD_t_ is OD of the aqueous phase recorded at 600 nm after 20 min.

#### 2.6.3. Mucin Adhesion Assay

The previously reported method was used to determine the in vitro mucin adhesion assay [45]. Flat-bottom 96 well microtiter plates were coated with 300 µL porcine mucin type III (10 mg/mL (Sigma-Aldrich, Inc. St. Louis, MO, USA) suspended in sterile PBS kept overnight at 4 °C. Plates were washed thrice with sterile PBS to remove unbound mucin from the wells. For competitive mucin adhesion, 100 µL of LAB culture with absorbance adjusted to 0.25 ± 0.05 and 100 µL of each of the *Salmonella* serovars were added to the mucin-coated wells simultaneously and were co-incubated for 90 min. After incubation, the wells were washed five times with PBS. Adhered bacterial cells were then treated with 300 µL Triton X in a sterile phosphate buffer solution to the separate bacterial cells. The viable cell count was measured by enumerating the LAB strains and *Salmonella* serovars on MRS agar, M17 agar, and SS agar. The % relative adhesion was calculated by (CFU/mL after adhesion/CFU/mL before adhesion) × 100.

#### 2.6.4. Biofilm Formation Potential of Lactic Acid Bacteria

The quantification of the biofilm formation by lactic acid bacteria was determined as described previously [21]. Briefly, 150 μL of MRS and M17 broth were added to each well of 96 flat-bottom microtiter plates along with 50 μL of an overnight culture of *L. reuteri* and *E. faecium*. The 96 well microtiter plates were incubated at 37 °C for 48 h. To quantify the biofilm formation, the wells were gently washed thrice with PBS. Biofilms were then fixed with 2 mL methanol for 15 min, and the microplates were dried at room temperature. Then 2 mL of the 2% *v*/*v* crystal violet solution was added to each well and was incubated at room temperature for 5 min. The excess stain was removed by washing with sterilized water. The adherent cells were removed with 2 mL of 33% *v*/*v* glacial acetic acid. Each well’s optical density (OD) was measured at 600 nm using a plate reader (Bio-Rad, Hercules, CA, USA). The experiment was performed in triplicate. The cut-off (OD_C_) was defined as the mean OD value of the negative control. On the basis of OD, isolates were classified as follows: (4 × OD_C_) < OD = strongly adherent, (2 × OD_C_) < OD ≤ (4 × OD_C_) = moderately adherent, OD_C_ < OD ≤ (2 × OD_C_) = weakly adherent, and OD ≤ OD_C_ = non-adherent [46].

### 2.7. Antimicrobial Potential Assessment

#### 2.7.1. Inhibition of Pathogenic Biofilm Formation

The effect of cell-free supernatant (CFS) of the LAB strains on the biofilm formation of *S*. Typhimurium and *S*. Enteritidis was evaluated as previously described [47]. The modified crystal violet assay was performed in 96-well microtiter plates for the biofilm quantification. First, 100 µL overnight-grown *Salmonella* strains were added in a well in the presence of 100 µL of pH neutralized CFS of the LAB isolates. The cultures were incubated at 37 °C for 48 h. The *Salmonella* test pathogens without CFS were used as a positive control. After incubation, the developed biofilm was washed three times with 200 μL of distilled water and was air dried. Then, 100 μL of 0.2% crystal violet (Merck KGaA, Gernsheim, Germany) was added to each well, and the plate was incubated for 20 min to allow for biofilm staining. Subsequently, the wells were washed three times with distilled water, and were air-dried and treated with 200 μL of 95% ethanol (Sigma-Aldrich, Inc. St. Louis MO USA) to dissolve the crystal violet crystals. The plate was incubated for 30 min, and the intensity of the crystal violet was measured at 600 nm using a microplate reader (Bio-Rad, Hercules, CA, USA). The ability of CFS to affect biofilm formation was calculated by comparing the absorbance values of the CFS treated wells versus the untreated control wells. The experiments were performed in triplicate. To estimate the reduction percent of CFS, the following formula was used:

Percentage inhibition = 100 − [(OD_600_ of wells in the presence of CFS × 100)/OD_600_ of wells in the presence of MRS]

#### 2.7.2. Scanning Electron Microscopy

Scanning electron microscopy (SEM) was used to identify any changes or ruptures in the cell morphology of the *Salmonella* due to the effect of the CFS of the LAB strains, as described previously [48]. The overnight-grown culture of both *Salmonella* serovars were treated with CFS of *L. reuteri* PFS4, *E. faecium* PFS13, and *E. faecium* PFS14, alone and in combination, in a sterile 24-well microtiter plate (Corning, Corning, NY, USA) with a 12-mm round cover glass and were incubated for 48 h at 37 °C. The overnight-grown culture of both *Salmonella enterica* serovars in a tryptic soy broth (TSB) media with 12-mm round cover glass was used as the control. After incubation, the microtiter plate was gently washed to remove the non-adherent cells before fixation with 2.5% glutaraldehyde in a potassium phosphate buffer (50 mM, pH 7). The cover glass was dehydrated by chilled ethanol in a series (*v*/*v*) ranging from 30, 50, 70, 90, and 100%. For SEM, the specimens were dried to the critical point, coated with gold, and photographed with a scanning electron microscope (JSM-IT500HR, JEOL, Akishima, Japan).

#### 2.7.3. Determination of the Antimicrobial Activity of the LAB Isolates

Aa time kill assay was performed as described previously [22]. *Salmonella* test pathogens were grown in a Luria broth (LB) media for 24 h at 37 °C. One hundred microliters of overnight-grown *S. enterica* serovars and pH neutralized CFS of LAB strains were co-cultured in a 96-well sterile microtiter plate and incubated at 37 °C. The number of viable bacteria was counted on an SS agar plate 0, 4, 8, 12, 24, and 48 h after incubation. *Salmonella enterica* strains without the cell-free supernatant of the LAB strains were taken as the positive control. The experiment was performed in triplicate.

### 2.8. Statistical Analysis

All of the experiments were carried out in triplicate. The standard deviations were determined with Microsoft Excel (Microsoft Corp., Albuquerque, NM, USA). A *t*-test was performed at the 95% confidence interval with SPSS20 (IBM Co., New York, NY, USA) to determine the statistical significance of the data.

## 3. Results

### 3.1. Bacterial Isolates

A total of 17 potential LAB strains were selected from the gut of broiler chickens. Among the 17 isolates, nine were chosen from MRS agar plates and eight were chosen from M17 agar plates. The isolates were identified as LAB based on the following characteristics: Gram-positive, catalase negative, non-motile, and cocci- or rod-shaped.

### 3.2. Safety Assessment

#### Hemolytic, DNase, and Antibiotic Susceptibility Assay

All 17 isolates showed negative results for hemolytic (γ-hemolysis) and DNase activity. However, a high resistance to most of the tested antibiotics, e.g., ciprofloxacin, chloramphenicol, third and fourth generation cephalosporin, amikacin, gentamicin, and vancomycin, was observed in the study (Table 1). Most of the strains were sensitive to rifampin, imipenem, meropenem, linezolid, and nalidixic acid. The criteria for selecting potential probiotics were as follows: isolates must be susceptible to clinically significant antibiotics, i.e., quinolones, ampicillin, amoxicillin-clavulanic acid, and cephalosporin [49]. Only three isolates, PFS4, PFS13, and PFS14, were chosen for further investigation based on the selection criteria.

### 3.3. Evaluation of Probiotic Properties

#### Tolerance to GIT Related Stresses

**Figure 1:**
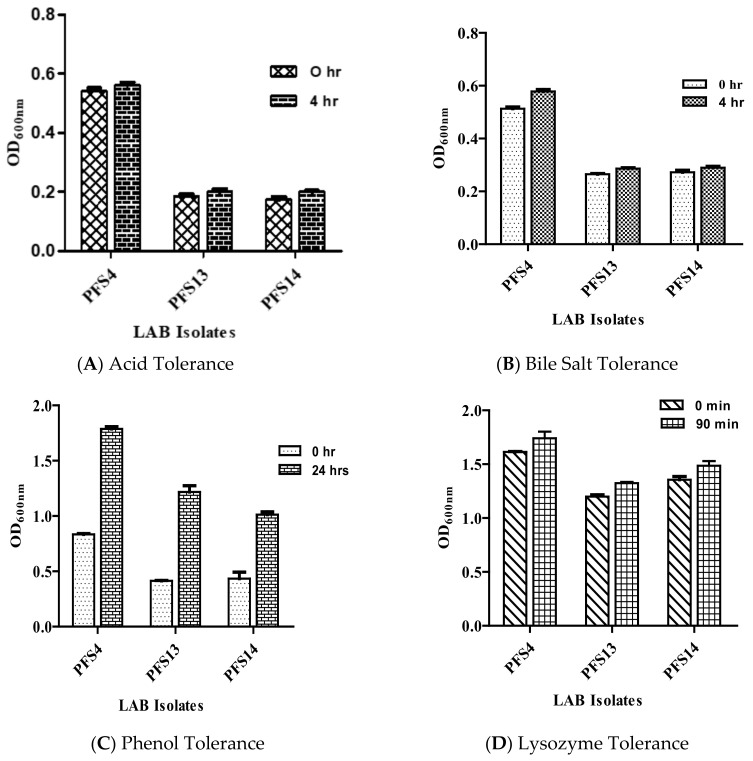
Survivability of probiotics in gastrointestinal tract (GIT) related stresses for LAB isolates: (**A**) acidic pH (pH 2) tolerance; (**B**) 0.3% bile salts tolerance; (**C**) phenol tolerance; (**D**) lysozyme tolerance. The standard error (*n* = 3 independent experiments) is indicated in the error bar.

The survival of isolates in the GIT is an essential feature of probiotics. All 17 isolates showed tolerance to GIT related stresses as shown in (Figure S1). After selection of three LAB isolates on the basis of their safety profile, the tolerance to GIT-related stresses of selected LAB strains was shown in (Figure 1A–D). Acid tolerance is a vital characteristic for all probiotics. Bile salt tends to damage the cell membrane structure, so tolerance to bile salt is an essential characteristic of probiotics. All LAB isolates survived to exposure of pH 2 and 0.3% bile for 4 h (Figure 1A,B). The results indicate that all of the tested strains showed a high resistance to acid and bile. These isolates were subsequently evaluated for phenol resistance, where these showed increased tolerance to 0.4% phenol with OD values >1.000 after 24 h of incubation (Figure 1C). All of the tested LAB isolates showed significant resistance to lysozyme (100 mg/L) after 90 min (Figure 1D).

### 3.4. Molecular Identification of LAB Strains

**Figure 2:**
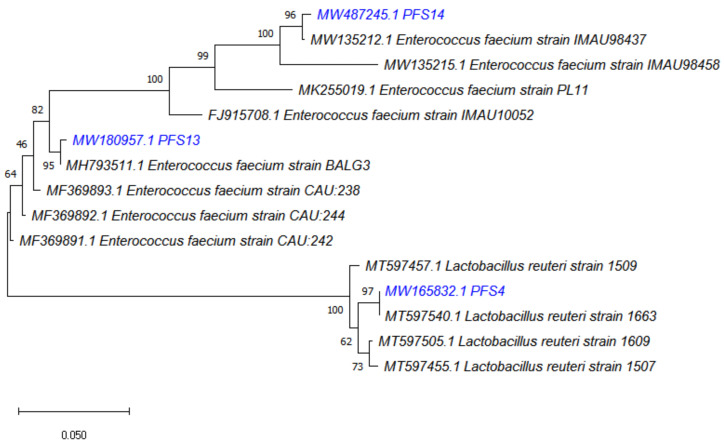
Phylogenetic tree showing the position of *L. reuteri* PFS4, *Enterococcus faecium* PFS13, and *Enterococcus faecium* PFS14 (in blue color) relative to the other phylogenetically close neighbors. Sequence alignment and phylogenetic inferences were obtained using the maximum likelihood method with MEGA X University Park PA, USA software. The numbers at the nodes are the percentages of bootstrap values obtained by repeating the analysis 1000 times to generate a majority consensus tree.

The 16S rRNA sequences obtained were compared using the public database of NCBI. As a result, the LAB isolates were identified as *L. reuteri* PFS4, *E. faecium* PFS13, and *E. faecium* PFS14. A phylogenetic tree of bacterial isolates was created based on the minimum evolutionary distance to obtain a well-resolved tree. It was found that isolate PFS4 was under the group of *Lactobacillus* spp. and was closely related to identified organism *L. reutei* strain 1663 with a strong similarity of a 97% branch value. Isolates PFS13 and PFS14 was under the group of *Enterococcus* spp. and were closely related to the identified organism *Enterococcus faecium* strain BALG3 and *Enterococcus faecium* strain IMAU98437, with a strong similarity of 95% and 96% branch value, respectively (Figure 2).

### 3.5. Cell Surface Hydrophobicity

**Figure 3:**
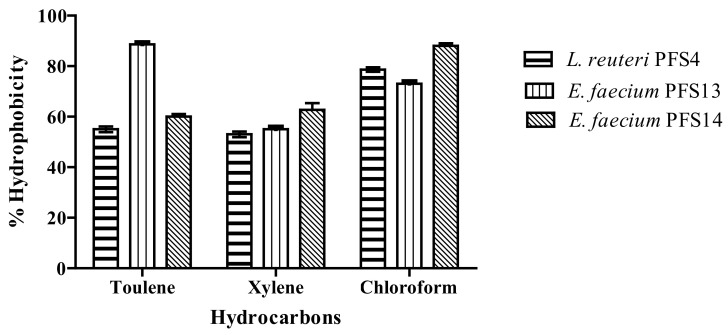
Percentage hydrophobicity of probiotic isolates against various solvents. Each value is the mean ± SD of three separate experiments.

The microbial adhesion hydrocarbon test indicates the adherence capability of probiotic strains to intestinal cells. It is an essential characteristic that helps probiotics colonize, control enteric pathogens, and modulate the host immune system. LAB isolates showed variable hydrophobicity towards xylene (53–64%), toluene (59–88%), and chloroform (78–89%) (Figure 2). *L. reuteri* PFS4, *E. faecium* PFS13, and *E. faecium* PFS14 showed a maximum affinity towards chloroform at 89%, 78%, and 83%, respectively. *E. faecium* PFS13 showed a high affinity (88%) towards toluene, while *L. reuteri* PFS4 showed less affinity (53%) towards xylene in (Figure 3).

### 3.6. Auto-Aggregation and Co-Aggregation Assay

Auto-aggregation helps with adherence to epithelial cells in the intestine, and thus provides competitive adhesion against gut pathogens. All of the strains tested in this study exhibited some degree of auto-aggregation, ranging from 71.2% to 80.1%. The results indicate that each strain showed a good auto-aggregation ability according to the criteria described by [43]. Among these probiotic strains, *L. reuteri* PFS4 isolates showed the highest auto-aggregation capacity (80.1 ± 3.1%) at 24 h.

The co-aggregation ability of selected LAB isolates with *Salmonella* depends on the serovars (Table 2). *E. faecium* PFS13 showed the maximum co-aggregation ability (66 ± 1.1% and 71 ± 1.5%) with *S.* Typhimurium and *S.* Enteritidis, respectively, while *L. reuteri* PFS4 and *E. faecium* PFS14 exhibited moderate co-aggregation with both *Salmonella* serovars. A combination of LAB isolates showed a high co-aggregation percentage (52.2% and 68.6%) with *S.* Typhimurium FML15 and *S.* Enteritidis FML18, respectively.

### 3.7. In Vitro Mucin Adhesion Assay

**Figure 4:**
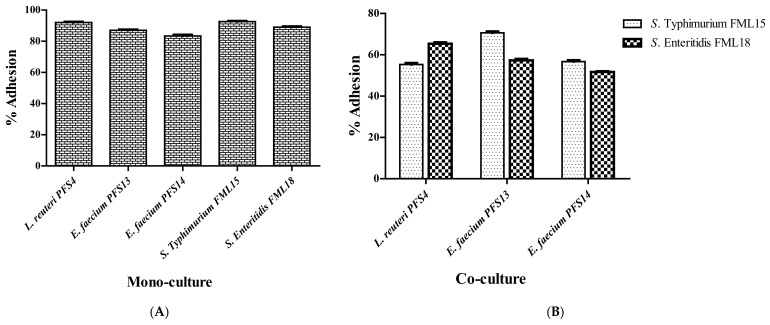
Percent adhesion values of the (**A**) monoculture of LAB strains and tested pathogens, and the (**B**) co-culture of LAB strains with tested *Salmonella* pathogens to porcine mucin Type III performed in microtiter plates. Each isolate’s relative adhesion (%) is calculated from the CFU values before and after the adhesion assay. Error bars represent the standard deviation of the trials.

All probiotic strains and *Salmonella* strains demonstrated a good mucin adhesion profile in the monoculture. The adhesion to porcine mucin films was >80% for all of the probiotic and *Salmonella* strains. The results revealed that *L. reuteri* PFS4 exhibited the highest percentage adhesion (92.6%), followed by *E. faecium* PFS13 (86.3%) and *E. faecium* PFS14 (84%) (Figure 4). However, the adhesion of *Salmonella* strains was significantly reduced in the presence of LAB isolates. The adhesion percentage of pathogenic strains, *S.* Typhimurium PF40 and *S.* Enteritidis PF76, was low (55.3% to 70.6%, and 51.6% to 65.3%, respectively) in the presence of LAB isolates compared with the untreated wells.

### 3.8. Biofilm Formation Assay

**Figure 5:**
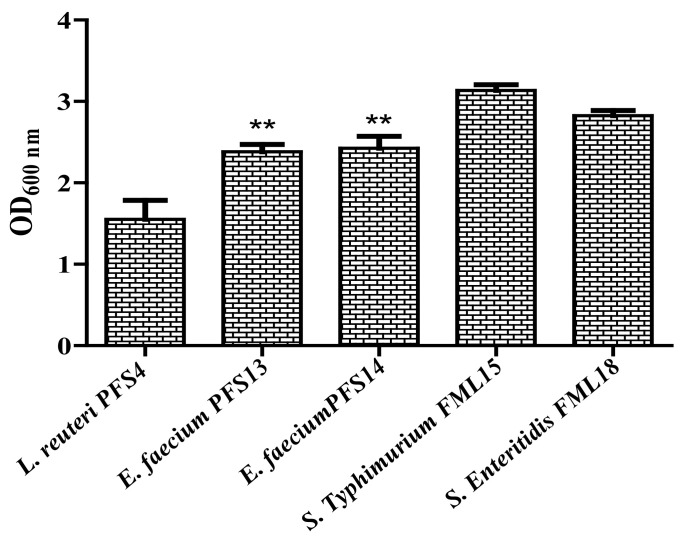
Biofilm forming abilities of LAB strains and the tested *Salmonella* pathogens (*S*. Typhimurium FML15 and *S*. Enteritidis FML18) evaluated using a crystal violet assay performed in a microtiter plate at OD 600 nm wavelength. Bars represent the mean, and error bars represent the standard deviation of three independent experiments. (**) Values show non-significance (*p* ≤ 0.05).

The biofilm formation by the probiotics is helpful in the competitive exclusion of gut pathogens in the intestine. Hence, the potential of probiotic candidates to form biofilms was evaluated in an in vitro assay. All of the probiotic candidate strains were capable of developing a moderate to strong biofilm (Figure 5). Both *E. faecium* strains exhibited a strong biofilm formation (OD > 2.0) potential, whereas *L. reuteri* PFS4 showed a moderate biofilm formation (OD < 2.0).

### 3.9. Effect of CFS on Biofilm Formation Ability of Salmonella enterica

**Figure 6:**
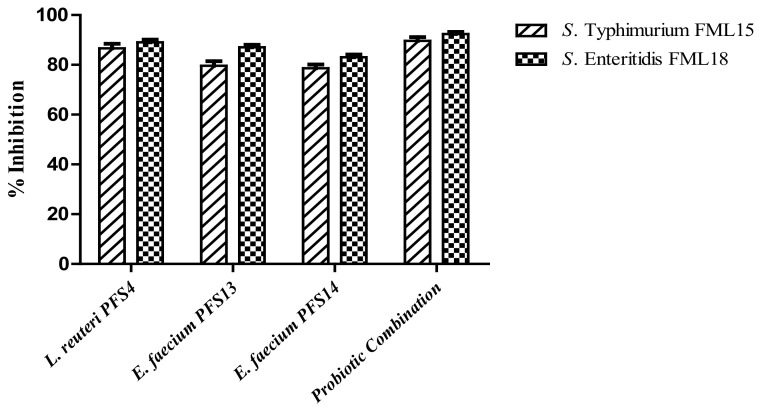
Percentage inhibition of biofilm formation of the tested *Salmonella* pathogens (*S*. Typhimurium FML15 and S. Enteritidis FML18) by pH neutralized CFS of LAB isolates evaluated by a modified crystal violet assay. Bars are representative of the mean, and error bars are representative of the standard deviation of three independent experiments.

The ability of probiotics to reduce *Salmonella* biofilms is an important characteristic for therapeutic probiotics post *Salmonella* infection. Therefore, the *Salmonella* biofilms were treated with pH neutralized probiotic CFS to assess the biofilm inhibition potential. Maximum inhibition was observed in the case of probiotic combination (90 and 92%) followed by *L. reuteri* PFS4 (87 and 89%), *E. faecium* PFS13 (80 and 87%), and *E. faecium* PFS14 (79 and 83%) for both *S*. Typhimurium and *S*. Enteritidis, respectively. The *Salmonella* biofilm was significantly inhibited (*p* ≤ 0.05) by the CFS of the three probiotics strains, alone or combined (Figure 6). However, the combination of CFS from all strains was better than the single strain CFS.

### 3.10. Scanning Electron Microscopic Study

**Figure 7:**
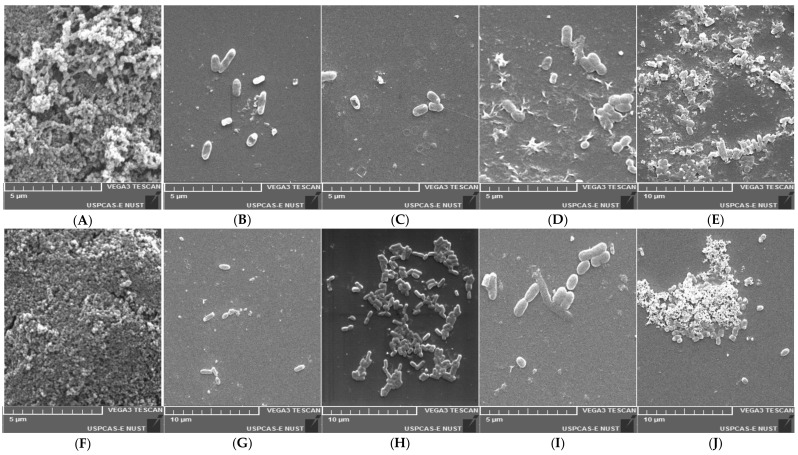
Scanning electron microscope images of the biofilm formed by *S. enterica* serovars Typhimurium and Enteritidis after 48 h at 37 °C. (**A**,**F**) Positive biofilm of *S*. Typhimurium and *S.* Enteritidis, respectively. (**B**,**G**) *S*. Typhimurium and *S.* Enteritidis treated with CFS of a probiotic combination, respectively. (**C**,**H**) *S*. Typhimurium and *S.* Enteritidis treated with CFS of *L. reuteri* PFS 4, respectively. (**D**,**I**) *S*. Typhimurium and *S.* Enteritidis treated with CFS of *E. faecium* PFS 13, respectively. (**E**,**J**) *S*. Typhimurium and *S.* Enteritidis treated with CFS of *E. faecium* PFS 14, respectively.

The biofilm produced by *S. enterica* serovars was confirmed by SEM. It was observed that the control sample (*S. enterica* serovars cultured into sterile TSB) showed a high cell density and aggregation (Figure 7A,F). Compared with the control, the neutralizing CFS of the LAB strains reduced the adherence and aggregation of the tested *Salmonella enterica* strains after 48 h of incubation. These results agree with the findings of the crystal violet technique, which showed a significant reduction in bacterial adherence and biofilm formation (Figure 6).

### 3.11. Effect of CFS on the Growth Kinetics of Salmonella enterica Serovars

**Figure 8:**
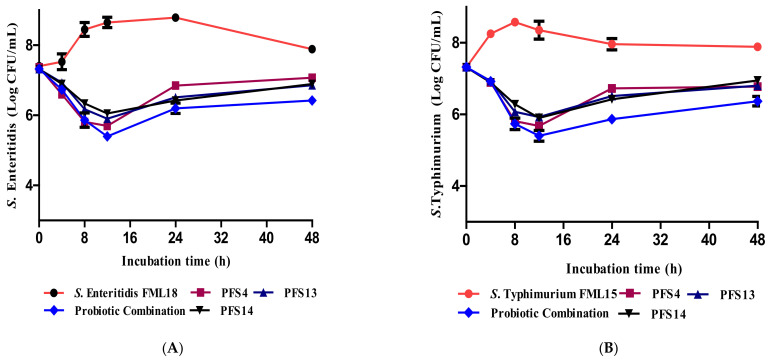
Growth kinetics of (**A**) *S*. Enteritidis FML18 and (**B**) *S*. Typhimurium FML15 monoculture and co-culture with neutralized CFS for three LAB strains alone and for the probiotic combination. Values are the means of triplicate experiments.

The results of the time-kill assay indicated the ability of CFS to reduce the growth of *Salmonella enterica* serovars (Figure 8). The growth of *S*. Typhimurium FML15 and *S*. Enteritidis FML18 was significantly inhibited after culturing with CFS in all of the tested time points compared with the control cultures incubated without CFS (*p* ≤ 0.05). The inhibitory effect was more prominent on both serovars, as indicated by the lower CFU, particularly after 12 h of incubation. The CFS of the LAB combination significantly reduced the count of both *Salmonella* serovars by 2 log compared with the control *Salmonella* without any CFS after 12 h of incubation.

## 4. Discussion

The emergence of multi drug resistant (MDR) *Salmonella enterica* serovars Typhimurium and Enteritidis constitutes a significant health and economic threat for poultry and humans [8]. Therefore, the application of probiotics in the poultry industry as a suitable alternative to antibiotics in order to control enteric pathogens has received tremendous attention in recent years [50]. Thus, we investigated potential probiotics from the chicken gut to control clinically significant antibiotic-resistant *Salmonella enterica*.

Initially, 17 isolates from 10 poultry fecal samples were isolated based on their colony morphology, Gram staining, and biochemical characteristics. However, only three strains, *L. reuteri* PFS4, *E. faecium* PFS13, and *E. faecium* PFS14, were retained based on the selection criteria. The findings were consistent with previous studies, which found *L. reuteri* [51] and *E. faecium* [52] in the chicken gut. According to the previous study, *L. reuteri* is the most common *Lactobacillus* species in the chicken GIT, primarily found in the crop and cecum [53]. In addition, many previous studies have reported on the protective effect of *L. reuteri* [54,55] and *E. faecium* [56] against *Salmonella* serovars and other gut pathogens.

Potential probiotic LAB strains should withstand digestive system stress to benefit the health of their hosts. The ability to tolerate acid, bile salts, lysozyme, phenol, and the production of antimicrobial agents are all good indicators of a strain’s survival in the GIT [57]. In this study, all 17 isolates tested for GIT-related stress tolerance exhibited a high potential to tolerate a low pH (2), 0.3% bile salt concentration, lysozyme (100 mg/mL), and 0.4 % phenol. The ability of LAB isolates to tolerate a low pH and bile is not a surprise, as one of LAB’s natural habitat is the gastrointestinal tract of humans or animals [58]. Our results are in agreement with previous findings that reported on the survival of different LAB strains, including *Enterococcus* spp., *L. salivarius*, *L. johnsonii*, and *L. reuteri*, isolated from healthy chicken gut, being able to survive at pH 2 and 0.3% bile concentration [59,60,61]. Another study reported the resistance of LAB strains to lysozyme and 0.4% phenol isolated from poultry gut at varying degrees [51].

*Lactobacilli* is a probiotic genus widely employed in animal production [62]. Most *Lactobacillus* species (*L. acidophilus*, *L. rhamnosus*, and *L. reuteri*) strains demonstrate no pathogenicity or acute oral toxicity in animals [63]. *Lactobacillus* are considered GRAS “Generally Recognized as Safe” because of their long history of safe use in animal feed [64]. However, some researchers reported some enterococcal species as opportunistic pathogens that can cause infections in humans and animals. Thus, determining the safety of these strains is critical for selecting these strains for industrial purposes and, more importantly, as probiotics. However, in several investigations, *E. faecium* has also been used as a probiotic candidate in poultry birds [65,66].

All of the initially screened 17 isolates showed negative results for a hemolytic DNase activity and demonstrated variable antimicrobial resistance patterns towards the tested antibiotics (Table 2). These results are following the previous study where *E. faecium* and *L. reuteri* showed a negative hemolytic and DNase activity [67]. The isolated LAB should be sensitive to antibiotics in order to avoid any potential transfer of undesirable antibiotic resistance to the intestinal microbiota [68,69,70]. Recently, many researchers have revealed that commensal microbes, including LAB isolates, contain antibiotic resistance genes similar to those found in pathogens [71]. Genes conferring resistance to tetracycline and cephalosporin detected and characterized in the *Lactococcus* spp. and *Lactobacilli* constitute a reservoir of resistance for potential food and gut pathogens, thus presenting safety issues [72,73]. These results are in agreement with previous findings [74,75,76], which reported the safety and sensitivity of *L. reuteri* and *Enterococcus* strains isolated from poultry gut towards common antibiotics. The high intrinsic resistance and susceptibility of probiotic microorganisms to a variety of antibiotics are quite significant. Some LAB species, including *L. reuteri*, *L. fermentum*, *L. rhamnosus*, *E. faecium*, *E. faecalis*, and *L. acidophilus*, have intrinsic resistance to vancomycin, sulfamethoxazole/trimethoprim, and nalidixic acid [77]. LAB with acquired/transferable drug resistance genes are not GRAS (generally recognized as safe) and should not be added to probiotic foods or animal feed. Conjugative plasmids typically carry antibiotic resistance genes that can be passed on to other bacteria, leading to antibiotic-resistant enteropathogenic bacteria [78]. Therefore, due to safety issues, the antimicrobial resistance pattern of the bacterial species is an essential criterion for their selection as probiotic candidates.

Bacterial adhesion to intestinal surfaces could be initiated by non-specific physical interactions such as hydrophobic interactions, followed by adhesion by specific cell wall components at a second level [79]. Microbial cell surface hydrophobicity (MATH) is the most commonly used in vitro method to assess cell surface hydrophobicity in lactic acid bacteria [80]. Some researchers have reported a correlation between hydrophobicity and adhesion [81]. The hydrophobicity test can be considered as a pre-test for probiotic bacteria’s ability to adhere to epithelial cells, preventing pathogens’ colonization (Falah et al., 2019). Hydrophobicity is an important property for enhancing the first contact between the bacteria and host cells. The hydrophobicity of the cell surface of *L. reuteri* and *E. faecium* is determined in order to know the cell surface properties responsible for aggregation and adhesion. In our study, LAB isolates showed variable hydrophobicity with different hydrocarbons (53–89%). Low to high hydrophobicity abilities were observed in *L. reuteri* LR11, LR19, and LR26 (30–71.10%) isolated from the poultry gut [60]. In addition, [82] obtained a high hydrophobicity (>80%) among *Enterococcus* spp. isolated from poultry. Some authors have considered that *L. reuteri* and *E. faecium*, with a high hydrophobicity, have a better ability to bind to epithelial cells [83]. Aggregation helps probiotic strains colonize in the intestine and significantly attaches intestinal epithelial cells, preventing *Salmonella* adhesion. This test is essential for the selection of probable probiotic bacteria from the gut [84].

Auto-aggregation allows microorganisms of the same species to form self-forming communities, and this process is most commonly associated with microorganisms adhering to the intestinal mucosa [85]. Our data revealed that LAB isolates showed a high auto-aggregation ability ranging from 71–80%. Similar findings were observed in previous studies, where *L. reuteri*, *E. faecium*, and other *Lactobacillus* spp. showed high auto-aggregation (70–90%) abilities [86,87]. In addition, some in vivo studies reported that the ability of high auto-aggregation of *L. reuteri* and *E. faecium* was strongly associated with adhesion [88,89].

Co-aggregation is the intercellular adhesion of different strains that are linked to the ability to associate with pathogens. The co-aggregation potential of gut probiotics enables the host to limit enteric pathogens like *S*. Typhimurium and *S*. Enteritidis [90]. In our study, *E. faecium* PFS13 had the most remarkable ability to co-aggregate with *Salmonella* Typhimurium FML15 and *Salmonella* Enteritidis FML18 (66.1% and 71.5%, respectively). Therefore, all three LAB strains in our study, with moderate to high co aggregation abilities, are potential probiotic candidates. The results obtained in our study correspond with previous findings, where *L. reuteri*, *Enterococcus* spp., and *L. acidophilus* showed a moderate to high co-aggregation (40–60%) ability with enteric pathogens, including *S. enterica* [42,91]. Many of the guidelines for probiotics’ health benefits regard their ability to stay viable and conform to the host’s intestine as a critical factor. Adherence allows probiotics to live longer in the GI tract and improves bacterial–host interactions, but it also aids in reducing gastric motility. As a result, probiotics’ ability to adhere to mucosal surfaces and epithelial cells is essential [92].

Bacterial adhesion to the host mucin is thought to be critical in helping probiotic species colonize in any setting, the ability to exclude food-borne pathogens competitively, and to improve their ability to stimulate the immune system. The mucus layer not only serves as the first physical barrier to bacterial invasion of the gut epithelium, but it also serves as the first point of contact for gut bacteria with host cells. For many pathogens like *Salmonella*, mucin adhesion is an important element in virulence in the host. [93]. The mucus of the small and large intestines may contain many receptors that mediate the adherence of beneficial or harmful microorganisms [94]. All LAB isolates in this study showed good adhesion (84–92.6%) to porcine mucin Type III. In a previous study, *L. reuteri* showed a better adhesion (74%) ability than other LAB strains to the intestinal mucin [95].

On the other hand, the adhesion of *Salmonella* serovars was significantly reduced when they co-cultured with LAB strains. The previous findings of an in vitro mucin adhesion assay revealed that *L. reuteri*, *L. rhamnosus*, and *L. casei* competitively excluded (13.26% to 25%) *S. paratyphi* A, *S.* Typhimurium SA2093, *S. flexneri*, and *S. aureus* TISTR from adhesion to porcine mucin. The inhibition of pathogen adhesion to the mucin is reported to prevent translocation and subsequently infection [96,97].

Pathogenic and non-pathogenic microorganisms form biofilms responsible for maintaining such microorganisms in healthy ecosystems in vivo [98]. The biofilm formation ability can be considered beneficial for probiotic strains, promoting colonization and long-term permanence on the host’s mucosa [99]. Our data showed that all three LAB isolates are strong biofilm producers. Moderate to strong biofilm production by *L. plantarum*, *L. reuteri*, *L. acidophilus*, *L. casei subsp. Pseudoplantarum.* and *E. faecium* have been observed in previous studies [23,100]. Scanning electron microscope (SEM) images demonstrated a strong adherence and aggregation of *S.* Typhimurium and *S*. Enteritidis bacterial cells. Our SEM images also showed that the adherence of cells was minimized after CFS treatment. Similar findings were observed in a previous study where the CFS of different probiotic strains reduced biofilm formation [22]. The results of the in vitro mucin adhesion assay, aggregation abilities, cell surface hydrophobicity, and biofilm formation capability of our LAB isolates showed strong adhesion abilities. They can prevent NTS in poultry gut through a competitive adhesion mechanism. Some of the *Lactobacillus* spp. with a low adhesion and co-aggregation ability were excluded in a previous study, as they could not be selected as potential probiotic candidates [101]. Our findings provide the foundation for understanding *L. reuteri* and *E. faecium* adhesion mechanisms and for predicting adherence in various host models. The authors of [102] revealed that *S.* Enteritidis growth in poultry birds was considerably reduced by *L. reuteri,* with a good coaggregation ability and mucin adhesion profile.

Lactic acid bacteria produce various antimicrobial agents, including organic acids, ethanol, diacetyl, hydrogen peroxide, and antimicrobial peptides [103,104]. The inhibition of pathogenic bacterial biofilms through probiotics is an attractive target for therapeutic intervention [105]. Probiotics have received significant attention in recent years, leading to the discovery of biofilm inhibitors to counter foodborne pathogens, including *S.* Typhimurium and *S.* Enteritidis [42,106]. Our data suggest that pH neutralized CFS of selected LAB strains alone and in combination significantly reduced the biofilm formation (up to 84%) of *Salmonella* strains compared with the control. The anti-biofilm property of CFS of *L. reuteri* and *E. faecium* against *Salmonella enterica* has been reported in previous studies [23,42,106]. The antagonistic activity of certain microbes and their extracellular antibacterial compounds found in cell-free supernatants (CFSs) provides an excellent potential for controlling foodborne pathogens, including *E. coli* and *Salmonella enterica* [107]. The findings of the growth kinetic study revealed that all three LAB isolates, individually and in combination, significantly reduced the growth of *Salmonella* test pathogens for up to 12 h. These results of the antagonistic activity against *Salmonella* could be due to various antimicrobial substances produced by probiotic strains. *L. reuteri* and *E. faecium* produced various antimicrobial substances such as lactic acid, acetic acid, hydrogen peroxide, bacteriocins, and bacteriocins, like inhibitory substances. The authors of [108,109,110,111] studied the efficacy of CFS on MDR *Salmonella* and other *Enterobacteracae* species and revealed that CFS significantly inhibits the growth of pathogens.

## 5. Conclusions

Based on our findings, we may infer that the LAB strains isolated from poultry gut exhibit endurance to GIT related stress. *L. reuteri* and *E. faecium* strains demonstrated a co-aggregation potential and competitively reduced the mucin adhesion of *S*. Typhimurium and *S*. Enteritidis. This study provides evidence that isolated probiotic strains from poultry gut have an in vitro antagonistic activity towards extended spectrum cephalosporin and fluoroquinolone (key antibiotics for salmonellosis) resistant *Salmonella* serovars. Therefore, *L. reuteri* PFS4, *E. faecium* PFS13, and *E. faecium* PFS14 can be potential candidates to control extended spectrum cephalosporin and fluoroquinolones resistant *Salmonella* serovars after being subjected to in vivo studies. In vivo studies using these strains can be performed to elucidate their safety profile, growth, and disease prevention ability. As part of the One Health approach, resistant-specific pathogen control can help mitigate the resistant pathogen load in animals and it’s spread to the associated food ecosystem.

## Figures and Tables

**Table 1:** Antibiotic susceptibility profile of the LAB strains isolated from the poultry gut.

Isolates	CN	CIP	K	VA	C	STM	IPM	MEM	NA	CFM	FEP	LZD	SXT	RIF	AK	TE	AMC
PFS1	R	R	R	R	S	R	S	S	S	R	R	S	R	S	R	R	S
PFS2	R	R	R	R	S	R	S	S	S	R	R	S	S	S	R	S	S
PFS3	R	S	R	R	R	R	S	I	S	R	R	S	R	S	R	S	I
PFS4	S	S	R	R	S	R	S	S	S	S	S	S	S	S	S	S	S
PFS5	R	S	R	R	R	R	S	S	S	R	R	S	R	S	R	R	S
PFS6	R	R	R	R	S	S	S	S	S	S	R	S	R	S	S	S	I
PFS7	R	S	R	R	R	R	S	S	S	R	R	S	R	S	R	R	I
PFS8	R	R	R	R	S	R	S	S	S	R	R	S	I	S	R	S	S
PFS9	R	S	R	R	I	R	S	S	S	R	I	S	I	S	R	R	R
PFS10	R	S	R	S	S	R	S	S	S	R	I	S	I	S	R	S	I
PFS11	S	S	R	S	S	R	S	S	S	R	R	S	R	S	R	S	R
PFS12	R	S	R	S	R	S	S	S	S	R	R	S	I	S	R	S	S
PFS13	S	S	R	S	S	S	S	S	S	S	S	S	S	S	S	R	S
PFS14	S	S	R	S	S	R	S	S	S	S	S	S	S	S	S	S	S
PFS15	S	R	R	R	S	R	S	S	S	R	R	S	R	S	R	R	S
PFS16	S	S	R	R	S	R	S	R	S	R	I	S	I	S	R	I	S
PFS17	S	S	R	S	S	R	S	I	S	R	R	S	I	S	R	I	S

Abbreviations: C—chloramphenicol (30 μg); AMC—amoxicillin-clavulanic acid (10 μg); CIP—ciprofloxacin(10 μg); CN—gentamicin (10 μg); SXT—trimethoprim/sulfamethoxazole (25 μg); K—kanamycin (30 μg); MEM—meropenem (10 μg); IPM—imipenem (10 μg); FEP—cefepime (30 μg); CFM—cefixime (5 μg); LZD—linezolid (30 μg); NA—nalidixic acid (30 μg); VA—vancomycin (30 μg); RIF—rifampicin (30 μg); Ak—amikacin (10 μg); TE—tetracycline (30 μg); STM—streptomycin (5 μg); R—resistance; S—sensitivity; I—intermediate.

**Table 2:** Auto and co-aggregation of potential LAB strains isolated from poultry.

Auto-Aggregation (%)		Co-Aggregation (%)	
Strains		*S*. Typhimurium FML15	*S*. Enteritidis FML18
*L. reuteri* PFS4	80.1 ± 3.1 *	39.3 ± 4.1	42.1 ± 1.1 *
*E. faecium* PF13	71.2 ± 2.1	66.5 ± 1.1 *	71.4 ± 1.5 *
*E. faecium* PF14	76.2 ± 1.8 *	40.1 ± 1.8 *	43.5 ± 1.1
Probiotic combination	61.1 ± 0.8	52.2 ± 2.3 *	68.6 ± 1.1 *

Values are means ± SD of the triplicate independent experiment. (*) values are significantly different (*p* < 0.05).

## Data Availability

The 16S rRNA sequences have been deposited in NCBI under the accession numbers; MW165832, MW180957 and MW487245.

## Supplementary Materials

The following are available online at https://www.mdpi.com/article/10.3390/ani11123435/s1, Figure S1: Survivability of gastrointestinal tract (GIT) related stresses of 17 LAB strains (**A**) Acidic pH (pH 2) tolerance. (**B**) 0.3% Bile salts tolerance. (**C**) Phenol tolerance. (**D**) Lysozyme tolerance.
