# Supplementary material for: Lactobacillus reuteri and Enterococcus faecium from Poultry Gut Reduce Mucin Adhesion and Biofilm Formation of Cephalosporin and Fluoroquinolone-Resistant Salmonella enterica
Source: Animals (Basel). 2021 Dec 1;11(12):3435. doi: 10.3390/ani11123435 (PMC8697943; doi:10.3390/ani11123435)
Supplement: Supplementary file 1 [file animals-11-03435-s001.zip › animals-1347658-SI.pdf]

SUPPLIMENTARY DATA

A) Acid tolerance

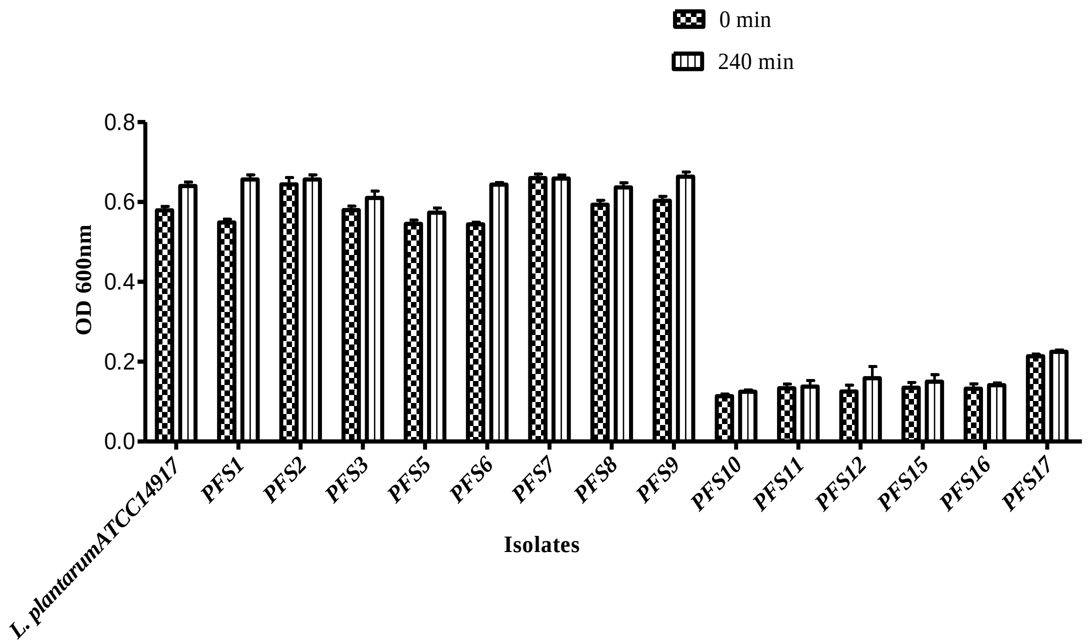

B) Bile tolerance

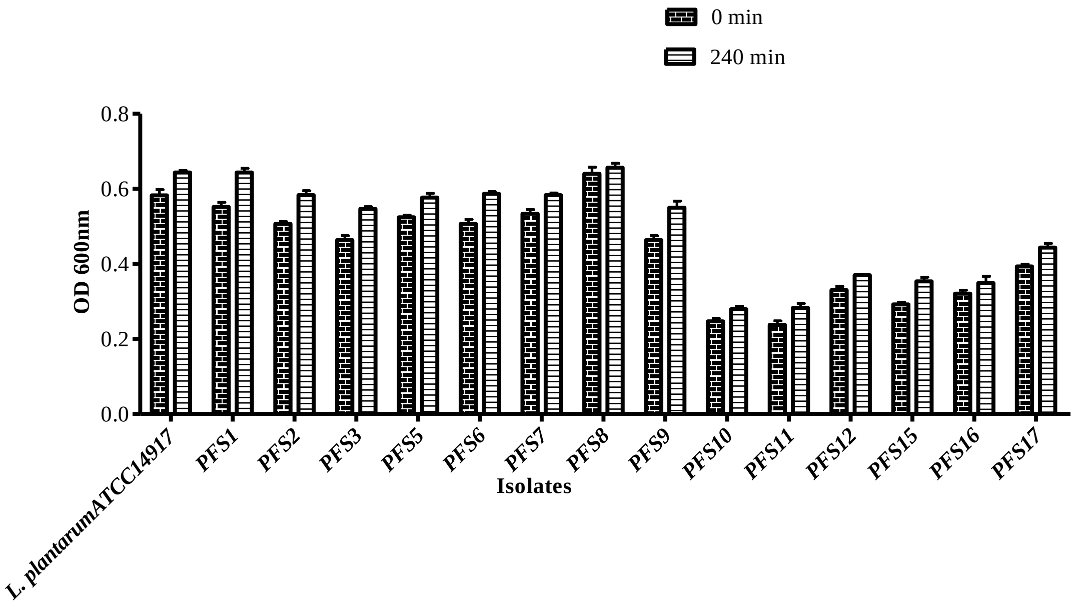

c) Phenol tolerance

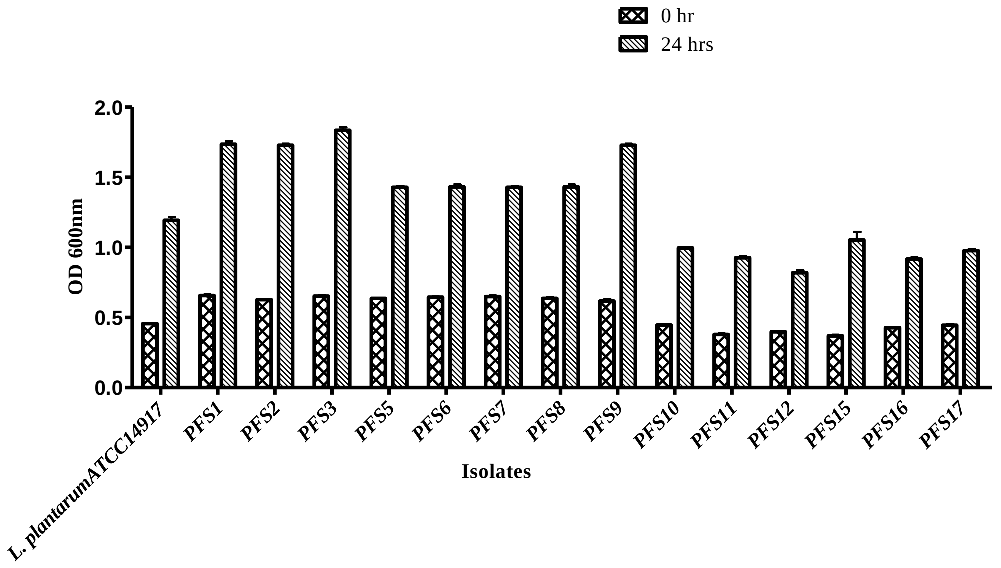

D) Lysozyme tolerance

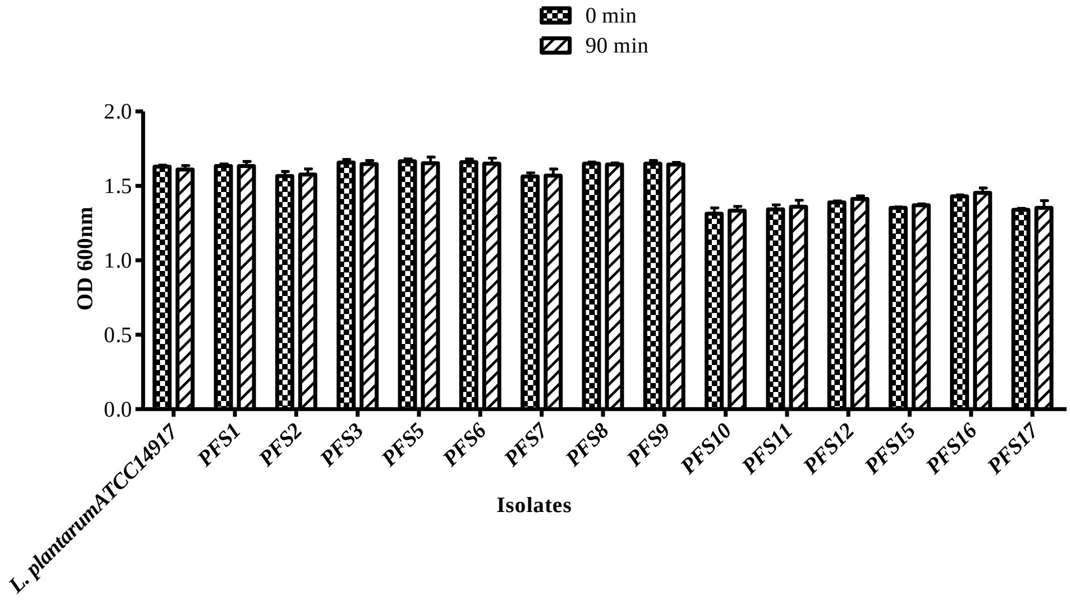

Figure S1. Survivability of gastrointestinal tract (GIT) related stresses of LAB strains **(A)** Acidic pH (pH 2) tolerance. **(B)** 0.3% Bile salts tolerance. **(C)** Phenol tolerance**. (D)** Lysozyme tolerance. Standard error (n = 3 independent experiments) is indicated in error bar. whereas control is *L. plantarum* ATCC 14917
